# Key decision-making factors for human papillomavirus (HPV) vaccine program introduction in low-and-middle-income-countries: Global and national stakeholder perspectives

**DOI:** 10.1080/21645515.2022.2150454

**Published:** 2022-12-09

**Authors:** Dominique Guillaume, Dur-e-Nayab Waheed, Meike Schlieff, Kirthini Muralidharan, Alex Vorsters, Rupali Limaye

**Affiliations:** a International Vaccine Access Center, Johns Hopkins Bloomberg School of Public Health, Baltimore, MD, USA; b Jhpiego, A Johns Hopkins University Affiliate, Baltimore, MD, USA; c School of Nursing, Johns Hopkins University, Baltimore, MD, USA; d Center for the Evaluation of Vaccination, University of Antwerp, Antwerp, Belgium; e HPV Prevention and Control Board, University of Antwerp, Antwerp, Belgium; f Department of International Health, Johns Hopkins Bloomberg School of Public Health, Baltimore, MD, USA; g Department of Health, Behavior & Society, Johns Hopkins Bloomberg School of Public Health, Baltimore, MD, USA; h Department of Epidemiology, Johns Hopkins Bloomberg School of Public Health, Baltimore, MD, USA

**Keywords:** Decision-making, human papillomavirus vaccination, low-and-middle-income countries, policy, HPV vaccination decision-making, HPV

## Abstract

Low-and-middle-income countries (LMICs) experience a high burden of cervical cancer. The human papillomavirus (HPV) vaccine prevents high-risk strains of HPV that cause cervical cancer; however, the integration of HPV vaccines into national immunization programs within many LMICs has been suboptimal. Our study evaluated key factors that drive the decision-making process for the implementation of HPV vaccine programs in LMICs. Stakeholder analysis and semi-structured in-depth interviews were conducted with national and global stakeholders. Interview data were analyzed through qualitative descriptive methods. Findings from our study revealed the decision-making process for HPV vaccines requires the involvement of multiple institutions and stakeholders from national and global levels, with decision-making being a country-specific process. Partner considerations, locally driven processes, availability of data, and infrastructure and resource considerations were found to be critical factors in the decision-making process. Future programs should evaluate the best approaches for investing in initiatives to enhance coordination, ensure vaccine introduction is locally driven, increase the availability of data needed for decision-making, and equip countries with the necessary resources to guide country decision-making in the face of increasingly complex decision-making environments.

## Introduction

Cervical cancer, which is primarily caused by human papillomavirus (HPV), is the fourth most frequent cancer among women globally and the leading cause of cancer-related deaths among women in low- and-middle-income countries (LMICs).^1,2^ The HPV vaccine has been commercially available for more than a decade and protects against high-risk strains of HPV that cause approximately 91% of cervical cancer cases.^3,4^ Since 2012, Gavi, the Vaccine Alliance, has subsidized HPV vaccines for girls ages 9–14 years old to make these vaccines accessible in LMICs.^3,5^ In 2020, the World Health Organization (WHO) established a global strategy for cervical cancer elimination. This strategy includes ambitious 90-70-90 targets to be achieved by 2030, with goals of 90% of girls fully vaccinated against HPV by 15 years of age, 70% of women screened for cervical cancer, and 90% of women diagnosed with pre-cancerous lesions to receive treatment and 90% of women with invasive cancer managed.^1,6^

Ensuring that HPV vaccines are available and accessible in LMICs that experience a disproportionate burden of cervical cancer will be crucial to meeting WHO cervical cancer elimination targets. Cervical cancer costs to health systems and families are substantial.^7-9^ Extensive research has been conducted on health system, programmatic, and supply-side challenges to new vaccine introduction, including HPV vaccines.^10-14^ A limited but growing body of research has also been conducted on factors that influence the decision-making process for HPV vaccine introduction.^15^ The decision-making process for vaccine introduction, vaccine type, and dosing schedules has become increasingly complex. This can be attributed to several factors, including a growing number of vaccine-preventable diseases, increasing diversity in vaccine products and technologies, greater programmatic complexity in dynamic environments, large volumes of global data and limited availability of local data, growing concerns regarding vaccine confidence and decision-making transparency, and challenges brought on by the COVID-19 pandemic.^16,17^ Compared to other routine vaccination programs, introducing the HPV vaccine has been described as a unique intervention with substantial challenges related to the target age group and the resources required for delivery.^15,18,19^ As many LMICs face health challenges that may impact the decision to introduce HPV vaccines, it is imperative to understand the decision-making processes behind the implementation of HPV vaccination programs.

Stakeholder decision-making is a crucial component of the implementation of HPV vaccination programs, though the specific factors that drive the decision-making process have not been well-articulated.^16^ This qualitative study aimed to identify and elucidate key factors of the decision-making process for the implementation of HPV vaccines in LMICs.

## Methods

### Country identification

We sought to include LMICs that had introduced or planned to introduce HPV vaccination at the national level within Africa and Asia to focus on context-specific lessons learned applicable across similar settings. To identify countries that fit these criteria, we conducted a mapping exercise to capture information such as geographical region, HPV vaccine introduction status, introduction date, dosing schedule, target age cohort, delivery strategy (campaign or routine delivery), and coverage. The programmatic information (i.e., target age cohort, delivery style, and dosing schedule) for each country was mapped in a table, and countries were divided into different regions (e.g., East Africa, West Africa, etc.). Sources including the International Vaccine Access Center’s VIEW-hub^20^ and the HPV Dashboard^21^ were used to list all countries in the WHO Regional Office for Africa (WHO AFRO), WHO South-East Asia Region (WHO SEARO), and WHO Western Pacific Region (WHO WPRO). Countries were ranked by diversity of HPV vaccination programs (e.g. single-age or multi-age cohort vaccination; dosing schedules; national subnational introduction), HPV vaccine coverage, and geographical representation, as well as HPV vaccine research found through database and gray literature searches.

### Identification of stakeholders

Once countries were selected, stakeholders from each country were identified using purposive sampling through literature searches on bibliographic databases, search engines, organizational databases (e.g., the International Papillomavirus Society, WHO, Gavi), gray literature from organizations working on HPV vaccination programs, and peer referrals. Stakeholders were grouped into two categories: national stakeholders and global stakeholders. National stakeholders included Ministry of Health (MOH) officials and stakeholders from Non-Governmental Organizations (NGOs) based at the country level. Global stakeholders consisted of academic researchers based in high-income countries (HICs) and LMICs with a history of conducting research relevant to HPV vaccine decision-making, introduction, and/or program implementation within LMICs, as well as global immunization partners, which included multilateral and bilateral organizations with active programs supporting HPV vaccines in LMICs (e.g., WHO, Gavi, International Agency for Research on Cancer, United Nations Children’s Fund [UNICEF], and Bill and Melinda Gates Foundation [BMGF]).

### Data collection

Semi-structured interview guides were developed through a comprehensive literature search evaluating studies that assessed decision-making and factors influencing vaccine introduction and scale-up in LMICs. The guide focused on decision-making around HPV vaccination. As the interviews were conducted and data emerged, the interview guides were revised. The research team met regularly throughout the data collection process to debrief and to revise interview guides as appropriate. Interviews were conducted from January 2022 through June 2022.

### Data analysis

Qualitative semi-structured interviews were conducted on Zoom and were audio recorded and transcribed. Transcripts were analyzed through an iterative process using thematic analysis. Inductive and deductive coding were employed, with codes developed both a priori from semi-structured interview guides as well as after transcript review. To develop an initial coding list, one transcript from each stakeholder category was randomly selected, and two authors independently performed open coding on each transcript. Open codes and corresponding text segments were then reviewed, and consistency of segmentation and code application were assessed.^22^ After open codes were agreed upon, the codes were then categorized into broader themes. A preliminary codebook was developed following the MacQueen et al. (1998) codebook structure guidelines and was used to analyze the remainder of the transcripts. The authors used an iterative process to adapt and revise the codebook as new codes emerged. The authors met regularly to discuss new codes, resolve disagreements, and revise the codebook.

## Results

A total of 110 stakeholders were contacted. Among the 31 who responded to participate in interviews, 18 were national stakeholders and 13 were global stakeholders. See Table 1 for information about global stakeholders and Table 2 for information about national stakeholders. Emerging themes included partner considerations, ensuring vaccine introduction is locally driven, data needed for decision-making, and infrastructure and resource considerations. An overview of the decision-making process is below, followed by elaboration on these themes. Challenges and recommendations in decision-making are summarized in Table 3.

**Table 1.** Global stakeholder categories.

Partner Category	Country	Expertise
Academic Partners (n = 7)		
	South Africa	Vaccinology
	South Africa	Vaccinology
	Kenya	Obstetrics and gynecology
	UK *	Vaccinology
	UK*	Economics and health systems
	Zambia	Health policy
	Nigeria	Immunology
Partner Category	Region	Expertise
Global Immunization Partners (n = 6)		
WHO	AFR, EUR	Vaccine preventable diseases
UNICEF	AFR	Health policy
GAVI	AFR	Health policy
BMGF	AFR	Health policy
IARC	AFR, EUR, AMR	Obstetrics and gynecology
PATH	AFR, SEAR, WPR	Immunization programming

Expertise of the global-level stakeholders interviewed for the study *Researchers based in the UK had ongoing or past programs of HPV vaccine related research in Rwanda and India.

**Table 2.** National stakeholder categories.

In-country stakeholders	Country	Expertise
National-level stakeholders (n = 10)		
	Cote d’Ivoire	National Immunization program and economics
	Ethiopia	National Immunization program and public health
	Kenya	National Immunization program
	Lao PDR	National Immunization program
	Rwanda	National Immunization program
	Zambia	National Immunization program
	Zimbabwe	Health promotion and policy
NGOs representatives (n = 8)		
	Cote d’Ivoire	National Immunization program
	East Africa Regional Representative	National Immunization and public health
	Kenya	Gynecology
	Lao People’s Democratic Republic	National Immunization program
	Zambia	National Immunization program
	Zimbabwe	National Immunization program

Expertise of the national-level stakeholders interviewed for the study.

**Table 3.** Challenges and recommendations in decision-making.

Themes	Challenges at the national level	Challenges at the global level	Actions
Decision-making process	Conflict on program placement at the national level creating competition on resources, resentment among stakeholders which leads to delay in decision-making process; EPI must obtain signoffs from multiple departments with this process varying from country-to-country;	Technical support for completion of Gavi application	Defining a clear pathway for decision-making process
Coordination among stakeholders	Difficulty in engaging different stakeholders due to time and workforce limitation at the national level	Difficulties in reaching a consensus from all stakeholders involved in the decision-making process; Lack of involvement of academic stakeholders and health care providers in decision-making	Explicit framework on engaging stakeholders and defining TORs Global partner support could be helpful
Transparent and participatory process/locally driven processes	Lack of clear framework on national stakeholders’ role	Discordance and lack of clarity on the specific partners to provide technical support; Global priorities and agendas that may not be in-line with country priorities	Defining roles among national partners to ensure ownership among stakeholders Dialogue between global and national stakeholders to ensure priorities correspond; Price transparency; Making vaccine data public within countries
Data for decision-making process	Lack of local data on disease burden delaying the decision making process at certain instances Lack of population data (i.e., number of girls eligible for vaccination) Lack of data available on serotype prevalence often required to support vaccine choice Inadequate human resource to capture data	Lack of costing data	Strengthening of data monitoring systems; Developing initiatives in collaboration with academic stakeholders to collect costing and population? data
Infrastructure and resource consideration		NITAGs are relatively weak which necessitates capacity support from global immunization partners; Resource mobilization; Support for countries that are not Gavi eligible	Heightening capacity support for NITAGs; considerations of financing programs for non-GAVI eligible LMICs

### Overview of the decision-making process

The decision-making process for the introduction of HPV vaccines involves different institutions and stakeholders from both the national and global levels, though decision-making is a country-specific process. Broadly, the prioritization of introducing vaccines into a country’s Expanded Programme on Immunization (EPI) is discussed by national bodies, such as the National Immunization Technical Advisory Groups (NITAGs). These national bodies are comprised of experts such as scientists and academics and are responsible for leading the prioritization of a country’s immunization program. Global immunization partners such as WHO, UNICEF, or Gavi may present evidence to NITAGs and discuss options and next steps for applying for the HPV vaccine. NITAGs evaluate the evidence and make recommendations to government stakeholders on whether to proceed with HPV vaccine introduction.

Once the decision is made to proceed with HPV vaccine introduction, the EPI initiates discussions with a technical advisory group such as the NITAG. The NITAG independently assesses several factors including disease burden, country capacity, and financial considerations before providing a recommendation. Once a NITAG is asked to advise on introduction of a vaccine, a smaller group of relevant experts is formed to discuss which specific vaccine should be introduced. After receiving the NITAG’s recommendation, the MOH and EPI engage various stakeholders to prepare a report. The EPI submits the report to the Interagency Coordinating Committee (ICC), which is comprised of the heads of various country-level organizations and global development partners, such as WHO, UNICEF, and Gavi. Once the ICC provides a recommendation, the EPI starts the Gavi application planning process.

**Figure 1.**
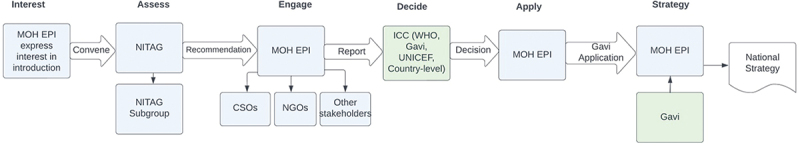
Global and national decision-making processes for HPV vaccine programs.

Global stakeholders noted that in many cases, EPI managers do not have executive decision-making power; they must conduct their own internal processes and require numerous departments and officials to sign off before they can make a decision. The specific departments and officials required to sign off on a recommendation vary among countries. After the decision to introduce a vaccine is made, stakeholders at the global level discuss how the vaccine will be introduced and establish a target date for introduction. Next, the country prepares its Gavi application. Gavi conducts full portfolio planning, or the planning process countries undertake to outline their long-term immunization goals, activities, and objectives. This plan is then used to inform a five-year National Immunization Strategy for the country. See Figure 1 for a summary of global and national decision-making processes.

## Key factors affecting decision-making for HPV vaccine program introduction

### Partner and coordination considerations

Several sub-themes emerged related to both national and global partner considerations, as well as coordination issues specific to each partner type.

#### Involvement of national partners in decision-making

Stakeholders involved in decision-making at the country level included the Ministry of Education, the Ministry of Finance, professional associations, and development partners/global partners. Various departments within the Ministry of Health were also involved, such as the Department of Maternal and Child Health, Department of Women’s Affairs, Department of Adolescent and Reproductive Health Services, Community Health Department, and the Department of Social Affairs.

##### National partners: coordination challenges

Country-level stakeholders stated that in-country coordination was a significant challenge. Countries experienced challenges with program ownership, as a respondent from an NGO explained:

At the beginning [before HPV vaccination introduction], in many countries, it was not clear who was in charge, and this did not facilitate decision making because I think there was sort of infighting: immunization program saying this is a vaccine, it belongs to us and the cancer control program, says, no, this is part of cervical cancer control. – NGO representative (National stakeholder)

National partners were in discussion with various international partners about vaccine introduction, creating difficulties in resource mobilization and delays in decision-making:

I think we’ve experienced this problem only with the HPV vaccine, and that is coordination. There have been numerous stakeholders in many countries, which was more of a barrier than an enabler. And then, let me also take it beyond the countries [in-country stakeholders] to international stakeholders. I think that different international stakeholders were approaching different programs, you know, to start discussions on HPV vaccination, others came through cancer control, others through sexual and reproductive health.- NGO representative (National Stakeholder)

This lack of coordination created a burden on already limited resources. Stakeholders voiced that the coordination challenges extended beyond decision-making and were also reflected in planning and implementation. Due to these challenges, adequate time was needed to get key stakeholders on board prior to introduction, as a MOH representative explained:

The strategic planning for HPV … [is]a bit conflicted in the sense that in one place you find HPV is part of the national cancer control strategy…in other places, you find that it is part of the adolescent health strategy or immunization program or clinical care because of the interest of the obstetricians and gynecologists at the beginning of the program, that’s part of why (numerous stakeholders competing for ownership) we delayed the discussions because these discussions started in 2012 and we could not introduce until 2019. Even when resources were mobilized by these four or five players, they tended to be spread quite thinly. - MOH representative (National stakeholder)

#### Involvement of global partners in decision-making

Global stakeholders were involved throughout the decision-making process in numerous roles including financing, data collection, presenting relevant data, advocacy, communications, and capacity building. Gavi’s pivotal role as a financial institution was well described, with stakeholders also noting that Gavi had numerous alliance partners who were essential for providing technical support to countries. Alliance partners were categorized as core partners (e.g., UNICEF, Centers for Disease Control and Prevention [CDC], WHO, World Bank, BMGF) and expanded partners (e.g., John Snow, Inc. [JSI], Jhpiego, PATH, Clinton Health Access Initiative [CHAI]). Core partners were essential in providing technical support to countries regarding field work, data collection, microplanning, developing Gavi applications, developing country plans, and providing training to health care workers. Expanded partners had a noteworthy role in decision-making and improving support and advocacy, especially CHAI, PATH, and Jhpiego. All global stakeholders reported the importance of Gavi:

Without Gavi, we wouldn’t even have access to the vaccine … it plays such a vital role in [providing access to the vaccine], but we need them [Gavi], to add more resources in this space. – Academic Researcher

##### Global partners: coordination challenges

Global stakeholders reported that in many countries, the decision to introduce vaccines is not controlled by the EPI manager. This is a significant barrier, as Gavi works with a country’s EPI manager to provide funding and EPI managers have their own internal process for approvals. These internal processes may include sign offs from multiple ministries such as the Ministry of Finance, Health, Women’s Affairs, Youth and Sports, and/or Education. The combination of these factors can create a difficult decision-making environment, according to a global immunization partner:

At the global level, there’s a naivete about the decision [to introduce HPV vaccines] … it is believed that] it is just the NITAG meets and the NITAG makes a recommendation. The recommendation’s endorsed, then the EPI manager is directed to make an application to Gavi…Gavi gives them the funding, the funding comes in and then they go on their merry way. But it’s not that simple …there are other [agencies] that need to be consulted…- Global Immunization Partner

Nearly all academic stakeholders said they were not involved in the decision-making process. Most academic stakeholders were both researchers and physicians who were concerned that researchers and health care providers were not present during decision-making. This perspective was mirrored by global immunization partners:

The [process] needs to involve people who are clinicians, oncologist, gynecologists, infectious disease specialists. They need to work together, create strong coalitions, great committees, and to get all the preparation together with them, and implementing preparatory activities together with them … because people trust clinicians when they say that this vaccine is good for your children, good for girls … because if something happened to the girl, then you go to clinician. You don’t go to immunization program. So that’s I think, one thing that didn’t happen in these countries. – Global Immunization Partner

### Locally driven process

The decision to introduce vaccines is a locally driven process influenced by factors such as vaccine prioritization, global pressure and influence, and transparency and stakeholder participation.

#### Vaccine prioritization

High disease burden, high mortality rates, availability of funding, and strong advocacy from local stakeholders were the primary facilitators of HPV vaccine prioritization:

It has become clear that cervical cancer is a big problem in many of our countries. Data have become available, and there’ve been many advocates; I think first ladies of the countries have played a very big role in raising awareness and women’s groups, as well as international NGOs in raising awareness – NGO representative (National Stakeholder)

While numerous data points were considered during the decision-making process, global stakeholders voiced that the introduction of HPV vaccines was highly political. Achieving political buy-in was crucial to support the decision to introduce.

I think there’s a layer of like politics that cross everything, but like, what’s the political agenda? Where’s the Minister in their appointment? Like, how does that impact [HPV vaccine prioritization]? What things, what strategies, what people are driving and what gets done versus [what does] not?- Global Immunization Partner

Competing health challenges and limited budgets resulted in governments having to decide whether to prioritize HPV vaccines over other long-standing health initiatives such as malaria, human immunodeficiency virus (HIV), and tuberculosis (TB) programs.

The HPV vaccination program is unlike routine infant immunization programs that the subcontinent has had long-standing experience with. It requires additional resources, additional capacity… this is often… the challenge. There are opportunities to explore external donor funding, but then while this might cover procurement on the vaccine itself, national governments are actually required to fund the actual program which is where majority of the cost is. And this has to be looked at in the context of the other health programs and public health issues that exist. - Academic Researcher

#### Global pressure and influence

External influence from global organizations played a pivotal role in shaping a country’s decision-making. Although decision-making was framed as a country-led process, numerous stakeholders, particularly global immunization partners, shared that countries felt pressured by demands from global organizations:

They’d like to say it’s the country’s decision-making but you don’t have to look no further than rota [rotavirus] and pneumococcal vaccine to see there was constant messaging being given from the global community and others that countries have to introduce new vaccines … you first you do your rotavirus vaccine, then you do your pneumococcal [vaccine], then you do your measles [vaccine}, second dose and now then Inactivated poliovirus vaccine (IPV). So, countries had to do that. Now it’s time for your HPV … when are you going to introduce HPV [vaccine]?…and these are the kinds of pressure mechanisms that happen from the global level… - Global Immunization Partner

The WHO cervical cancer elimination goals were also reported as a major facilitator in vaccine introduction.

It [global influence] is not always negative, of course, there were many countries, for example, which use the influential figures within the country in taking the issue in a wider women’s health or cervical cancer elimination agenda. So there are many good examples [of global influence] - Global Immunization Partner

#### Transparency and stakeholder participation

Both global and national stakeholders described the need for transparency during decision-making. Additionally, it was important that key stakeholders were involved throughout all stages of the process and had accurate information on decision-making procedures. There was an emphasis on ensuring that communities knew the decision to introduce HPV vaccines was not influenced by private-sector agendas and that adequate information was being disseminated to communities:

If your decision-making process is not transparent, not inclusive, and people think that the Minister of Health introduced vaccine because it was kind of influenced by manufacturers, you can make a wonderful campaign … it won’t work. You need to do everything right, starting from the decision making – inclusive, transparent, communicate to the public, why, what, and how we are going to introduce. Then, of course, the campaign itself should be based on evidence, you need to collect the evidence, you need to develop strategy, you need to implement the strategy. - Global Immunization Partner

A lack of transparency resulted in target communities’ reluctance to receive the HPV vaccine due to distrust. This also caused tension during the decision-making process, when there was miscommunication about which partner(s) would assist with countries’ technical needs after a Gavi application was submitted:

There also has to be sort of the willingness for … the decision makers to consider this evidence in a way that’s transparent….that’s evidence-based, … I think ideally, the process should be that the advisory body does its evidence, evaluation, and discussions in a way that’s very transparent, so that when the decision is made, it’s very clear what evidence they considered. And if they made a decision, contrary to advice, that’s fine. It’s their prerogative, but it’s very clearly explained why they did. – Academic Researcher

### Data availability

The decision-making process requires disease-specific data as well as local data collected by the countries themselves.

#### Local data requirements

Both in-country and global stakeholders reported the importance of having local data and generating local evidence through pilot/demonstration projects, with an emphasis on countries conducting their own assessments and leading data collection procedures.

Before introducing the vaccine, proceeding with a correct methodology to see what the cost of the introduction is [should occur]. We even often do pilot projects, like for HPV we did a demonstration project in two districts. After this demonstration project, we also have a post-introduction evaluation, to see what the level of acceptance is and we correct everything before being able to introduce. - MOH representative (National stakeholder)

Although countries prefer to have local data, this type of data is often unavailable, which delays the decision-making process. Even when local data is available, it is often not used.

The other issue at the subnational level is the use of local data for decision-making. But we note that data is not being used to inform actions at the district level so that they have targeted interventions where they identify challenges informed by their data. So usually, data may be used because they have to submit a report or they have to make a presentation because it’s that time of the year when they should show their performance – MOH representative (National stakeholder)

Being able to provide country-specific cost data was noted as essential:

We need to do more studies profiling, what happens at that level- what happens to that family unit when a mother dies? What is the cost of the system that that Minister of Treasury has to pay back? So, what’s the cost for them to help us deliver vaccines in the future? You do it now in 10 years’ time, we’re telling them that what you’re spending in radiotherapy, that cost will be gone. Number two, this woman didn’t die. And you will save this money …I do radiotherapy, that cost will be gone. Number two, this woman didn’t die. And you will save this money. I don’t know that we do enough of that [cost-effectiveness analysis]?.- Academic Researcher

#### Disease-specific data

In-country stakeholders reported that the decision-making process is often based on data related to disease burden and mortality.

I think data is always at the top because data must be available, and this must be perceived as a public health problem before the policy discussions can be held. So, availability of data [local data] has always been a challenge in some of these countries, and most of the countries increasingly want to use local data [for decision-making process] – NGO representative (National stakeholder)

However, local disease burden data are often unavailable, especially data on disease and serotype prevalence. Different countries evaluate different inputs during the decision-making process.

There are different inputs that the countries evaluate, first of all, the disease burden, of course, if this particular disease is a public health problem in the country … but that [disease burden] I think is lost its priority now, because the WHO recommendation for most of the new vaccines is universal … I mean, WHO recommends all countries to introduce particular vaccines, including HPV, so, that is less of an issue.- Global Immunization Partner

#### Infrastructure and resource considerations

At the global level, the decision-making process includes consideration of several factors related to infrastructure and resources including health system capacity, role of advisory bodies, and financing and budget allocations.

#### Health system capacity

Stakeholders articulated implementation challenges related to health system infrastructure and coordination. Global stakeholders described how vaccine packages and delivery platforms are selected. As health systems in many LMICs aren’t equipped for the needs of adolescents, many countries selected schools as the primary delivery platform for HPV vaccines.

But then, once the countries make a decision…if you look at the general vaccination programs in the LMICs, vaccination program is one of the most successful programs in LMICs. If you look at the HPV vaccine, in the context of HPV vaccination, if you look at the first dose coverage, first dose coverage in the LMICs is actually better than what it is in the high-income countries. So, the vaccination programs are really very efficient … but then they need to have the vaccine, they need to have the appropriate guidelines that they can employ to implement it. – Global Immunization Partner

Another important factor in the decision-making process was taking a comprehensive approach and introducing HPV vaccines with other health services like cervical cancer screening programs and HIV programs.

I think what is extremely important is that we also collaborate within WHO with our cervical cancer program, because when we come to the country, and advocate for the introduction, HPV vaccine, it’s always done within the comprehensive programs that should address cervical cancer screening as well as cervical cancer treatment and care. – Global Immunization Partner

#### Role of advisory bodies

Stakeholders had mixed views on the role of advisory bodies in the decision-making process. Global immunization partners (e.g., PATH, UNICEF, WHO) prioritized the importance of NITAGs, whereas national stakeholders did not perceive NITAGs as having the same level of importance in the process:

NITAGs are actually quite weak. And they are also only advisory bodies. There are other [agencies] that need to be consulted and sort of have political buy in, in order for the Ministry of Health to feel confident that they want to make an application to Gavi, because as you might know, they have to put some of their own money at the table. NITAGs is not the end all, because some of them are so weak, they can make a recommendation, but it doesn’t have to be adhered to…. - Global Immunization Partner

In some countries, NITAGs do not have the capacity to build strong cases for introduction, which has contributed to delays in decision-making. Even when NITAGs were presented with data, it was not always sufficient to support decision-making:

We’ve presented evidence to the NITAG four times over the last six years, and they have yet to make a decision or recommendation for HPV vaccine. Instead, they keep asking for more and more data … the recent one they wanted was a cost effectiveness analysis. So, we got funding to do a cost-effectiveness analysis for them. We did it- we presented the results, they still made no decision and no recommendation to government. And that’s where it stands today … That’s a conversation that’s been going on for almost eight years. - Global Immunization Partner

#### Financing and budget allocation

Gavi grants are meant to partially fund a program’s launch and introduction, with countries responsible for financing their own maintenance budgets. Countries applying for Gavi support are aware of their co-payment cost. They also complete an itemized five-year budget that indicates what items will be paid for by technical partners. Stakeholders provided examples of financial coordination challenges among technical partners:

‘You know how partner money comes? So, Government will have some money and partners will come with money at the table, but most partner money will be tied to particular objectives, so they are objectives that may fall in what I would refer to as ‘gray areas.’ So that was one of the challenges; trying to mobilize those particular resources for those particular gray areas that would help in terms of the support. I think that’s one of the biggest challenges. So trying to see who had more of a flexible budgets to move money from one pot to the next. - NGO representative

However, governments often state they will pay for certain line items in their budget but then later claim they are unable to pay for those items; they will subsequently ask partners to finance those activities. Additionally, as HPV vaccines are relatively expensive and their delivery is complex, they have been downgraded in certain countries in favor of other vaccines. Stakeholders also mentioned the cost of running programs every year, the untraditional delivery targets, and the delivery complexity as considerations in decision-making. For countries that were not eligible for Gavi support, cost and complexity were significant deterrents to introduction:

I don’t know that [cost-related factors] are as well understood… in terms of the decision-making process at country level, like, you know, ‘what it’s gonna cost? what Gavi will provide?’ … A lot of times countries will develop a budget, and they’re making estimates about how to design their program and what it will take. And a lot of the time, what I’m learning is it’s actually the budgets are coming in over what the Gavi introduction grant will cover, which is also just a signifier that this program is more expensive. - Global Immunization Partner

While vaccine pricing information is publicly available, global stakeholders reported that it is not provided by all countries:

Countries share information about the price, what they pay for HPV vaccine through WHO reporting. And this information is available on the web platform. Unfortunately, not all countries provide this information, they do not provide it regularly. And I think improving this information sharing about the prices would also help.- Global Immunization Partner

## Discussion

Our study is one of the first to provide an understanding of the decision-making landscape for HPV vaccine introduction from both global and national perspectives. Findings from our study revealed that partner considerations, encouraging locally driven decision making, data availability, and infrastructure and resource considerations are critical factors in the decision-making process. Prior research has also highlighted the importance of these factors in driving decision-making for vaccine introduction across different antigens, including HPV. For instance, among numerous studies evaluating the introduction of various vaccines, disease burden was noted as a universal driver in vaccine adoption and introduction.^23-27^ This corroborates our findings that stakeholders often rely on disease burden data to drive HPV vaccine introduction.

Availability of local data was found to be imperative to support decision-making, with local data preferred over neighboring country data, which has also been reported in other studies.^11, 27-29^ As the amount of information on vaccines and immunization programs increases, a heightened focus should be placed on strengthening health information systems and local capacity to collect locally generated evidence.^18^ Currently, stakeholders face information overload due to an increasing volume of global data, while simultaneously experiencing a lack of local data further compounded by barriers to access.^16^ The relatively higher price of HPV vaccines, challenges in meeting Gavi co-payments, and staffing costs can make it difficult to prioritize HPV vaccines in the face of numerous competing health challenges and limited resources within many LMICs. In the context of limited resources, positioning cervical cancer prevention through HPV vaccination as a justifiable investment is a considerable challenge.^18^ Furthermore, countries that have not yet introduced HPV vaccines must choose to invest in either vaccines or screening programs and decide whether the two services can be integrated.^8,19,30^ Increasing capacity, considering alternate delivery platforms, and evaluating the costs of additional resources required to successfully reach preadolescents and adolescents have also been noted as critical in the decision-making process.^31-33^

In comparison to the introduction of traditional childhood vaccines, there are unique considerations in the decision to introduce the HPV vaccine. The selection of effective delivery platforms to promote HPV vaccination coverage among adolescent girls has remained a challenge in the implementation of HPV vaccination programs.^19,34,35^ A lack of focus on adolescent health in many LMICs has resulted in poorly developed adolescent health services, which poses a considerable challenge to the implementation of HPV vaccination programs.^19,30^ Overcoming these challenges mandates heightened collaboration and coordination between sectors at the national and global level. Coordination and communication among diverse stakeholders with their own agendas and priorities is a barrier to the decision-making process. Our study found an emphasis on ensuring that decisions were locally driven; similar findings have been reported in studies assessing decision-making for HPV and other antigens when national ownership of the vaccine introduction decision is paramount for the credibility and sustainability of immunization programs.^28,35^ While global immunization partners, NGO partners, and MOH personnel in our study were found to be highly involved in the decision-making process, academic stakeholders were largely excluded from decision-making and were typically informed about HPV vaccine introduction after the decision had been made. This raises concerns about decision-making transparency and may potentially lead to substantial barriers in successful vaccine introduction due to discrepancies in communication. Decisions to introduce HPV vaccines must involve all relevant stakeholders and must be locally driven, with an emphasis on capturing important country and contextual nuances to guide decision-making.^28,36^

## Limitations

Our study is not without limitations. While the authors developed a comprehensive strategy to ensure the identification of stakeholders for this study, it is possible that certain stakeholders were not identified due to limitations of the search strategy (i.e., missing key terms, not identifying all relevant databases). In addition, although the authors contacted 110 stakeholders, the response rate was fairly low, with several stakeholders lost to follow-up. This could potentially be due to the timing of our interviews, which were conducted between Jan 2022 – April 2022 when targeted stakeholders were heavily involved in COVID-19 related activities. The use of purposive sampling and the low response rate allow for the possibility of selection bias within our study. It is also possible that social desirability bias impacted participant responses. While our study covered a limited number of countries and had a relatively small sample size, the diversity we achieved in our sample resulted in information-rich cases providing several different perspectives on HPV vaccine decision-making.

## Conclusions

This study provides an understanding of factors that influence decision-making for introducing HPV vaccine programs in LMICs with high burdens of cervical cancer. While meeting WHO cervical cancer elimination goals has become a global priority, future programs should evaluate the best approaches for investing in initiatives to enhance coordination, ensure locally driven processes, increase available data, and equip countries with the necessary resources to guide country decision-making in an increasingly complex environment.

## Data Availability

Data were collected from participants with an agreement that their names and organizations’ names would not be shared publicly. Therefore, the data will be shared anonymously upon a reasonable request.

## Abbreviations

LMICs: Low-and-middle-income countries
HPV: Human papillomavirus
Gavi: Gavi, the Vaccine Alliance
WHO AFRO: WHO Regional Office for Africa
WHO SEARO: WHO South-East Asia Region
WHO WPRO: WHO Western Pacific Region
WHO: World Health Organization
MOH: Ministry of Health
NGOs: Non-Governmental Organizations
HICs: High-income countries
UNICEF: United Nations Children’s Fund
BMGF: Bill and Melinda Gates Foundation
NITAGs: National Immunization Technical Advisory Groups
EPI: Expanded Programme on Immunization
ICC: Inter-Agency Coordinating Committee
CDC: Centers for Disease Control and Prevention
JSI: John Snow, Inc
HIV: Human Immunodeficiency Virus
TB: Tuberculosis
IPV: Inactivated poliovirus vaccine
